# Rectal and Naris Swabs: Practical and Informative Samples for Analyzing the Microbiota of Critically Ill Patients

**DOI:** 10.1128/mSphere.00219-18

**Published:** 2018-06-13

**Authors:** Saumya Bansal, Jenny P. Nguyen, Aleksandra Leligdowicz, Yu Zhang, Kevin C. Kain, Daniel R. Ricciuto, Bryan Coburn

**Affiliations:** a Department of Laboratory Medicine and Pathobiology, University of Toronto, Toronto, ON, Canada; b University of Toronto, Interdepartmental Division of Critical Care, Toronto, ON, Canada; c University Health Network, Division of Critical Care Medicine, Toronto, ON, Canada; d Department of Medicine, University Health Network, Toronto, ON, Canada; e Department of Medicine, Division of Infectious Diseases, University of Toronto, Toronto, ON, Canada; f Lakeridge Health, Division of Infectious Diseases, Oshawa, ON, Canada; University of Kentucky

**Keywords:** 16S RNA, critical illness, human microbiome

## Abstract

Commensal microbiota are immunomodulatory, and their pathological perturbation can affect the risk and outcomes of infectious and inflammatory diseases. Consequently, the human microbiota is an emerging diagnostic and therapeutic target in critical illness. In this study, we compared four sample types—rectal, naris, and antecubital swabs and stool samples—for 16S rRNA gene microbiota sequencing in intensive care unit (ICU) patients. Stool samples were obtained in only 31% of daily attempts, while swabs were reliably obtained (≥97% of attempts). Swabs were compositionally distinct by anatomical site, and rectal swabs identified within-patient temporal trends in microbiota composition. Rectal swabs from ICU patients demonstrated differences from healthy stool similar to those observed in comparing stool samples from ICU patients to those from the same healthy controls. Rectal swabs are a useful complement to other sample types for analysis of the intestinal microbiota in critical illness, particularly when obtaining stool may not be feasible or practical.

**IMPORTANCE** Perturbation of the microbiome has been correlated with various infectious and inflammatory diseases and is common in critically ill patients. Stool is typically used to sample the microbiota in human observational studies; however, it is often unavailable for collection from critically ill patients, reducing its utility as a sample type to study this population. Our research identified alternatives to stool for sampling the microbiota during critical illness. Rectal and naris swabs were practical alternatives for use in these patients, as they were observed to be more reliably obtained than stool, were suitable for culture-independent analysis, and successfully captured within- and between-patient microbiota differences.

## Abstract (precis)

Perturbation of the microbiome has been correlated with various infectious and inflammatory diseases and is common in critically ill patients. Stool is typically used to sample the microbiota in human observational studies; however, it is often unavailable for collection from critically ill patients, reducing its utility as a sample type to study this population. Our research identified alternatives to stool for sampling the microbiota during critical illness. Rectal and naris swabs were practical alternatives for use in these patients, as they were observed to be more reliably obtained than stool, were suitable for culture-independent analysis, and successfully captured within- and between-patient microbiota differences.

## INTRODUCTION

Interactions of gut microbiota with human hosts are important modulators of disease biology (1, 2) and of potential value as novel diagnostic and therapeutic targets during critical illness (3–9). Dysbiosis, the pathological perturbation of the microbiota, typically characterized by overabundance or absence of key microbial community members (taxa), has been implicated in various inflammatory diseases (10–16). However, very few studies have been done to study the stability, resilience, and temporal dynamics of the microbiota in critically ill patients.

For microbiome data to be incorporated into human observational and experimental studies and, ultimately, into clinical decisions in cases of critical illness, standardized, high-throughput, and reliable methods for sampling the microbiota are needed (8). Sampling methods for the microbiota of critically ill patients should meet several criteria: (i) samples must be obtainable from patients when indicated for clinical research, including at daily or (potentially) hourly intervals; (ii) they must have sufficient microbial DNA to assay community composition without the typical optimal conditions commonly used for studies of healthy volunteers; (iii) they should reproducibly resolve microbiota differences within a patient over time, between patients, or between anatomical sites; and (iv) they should be practical indicators of disease-dysbiosis relationships relevant to the critically ill population.

In this study, we compared four sample types—rectal, naris, and antecubital swabs and stool—for analysis of the microbiota in a small cohort of critically ill patients admitted to an intensive care unit (ICU). We assessed the ease of acquisition, suitability for 16S sequencing, and correlation of the microbiota with plasma cytokines as a nonmicrobiological indicator of patient immune responsiveness for each of these sample types.

## RESULTS

### Patient population.

For our prospective comparison of multiple sample types from the same patients, 175 samples were obtained from 9 ICU patients (6 male and 3 female) with a median age of 68 years (range, 45 to 80) enrolled at a medical-surgical ICU at a community hospital in December 2015. They represented a heterogeneous ICU population with various active and past medical diagnoses (summarized in Table 1) and were enrolled consecutively. Two study participants, patients 1 and 3, had a prolonged ICU stay prior to enrollment in the study and were chronically ventilated but clinically stable throughout the study period. No plasma samples were collected for these participants as daily blood work was not routinely ordered. Three of nine subjects died within 28 days of study enrollment.

**TABLE 1:** Study subject characteristics and primary diagnosis^a^

Subject no.	Age (yrs)	Sex	ICU diagnosis	No. of study days of ICU stay	Status after 28 days
1	52	M	End-stage COPD	171–177	ICU
2	80	F	Postesophagectomy/chylothorax	21–26	Discharged
3	66	F	ALS	392–398	ICU
4	72	M	CAP/CHF	13–17	Dead
5	45	M	Multiple CVAs	21–27	ICU
6	68	M	End-stage COPD and CHF	36–40	Dead
7	80	M	Hemorrhagic stroke	7–13	ICU
8	57	M	Cardiogenic and septic shock, AKI	7–13	Dead
9	71	F	Chronic renal failure and heart block	20–26	ICU

a AKI, acute kidney injury; ALS, amyotrophic lateral sclerosis; CAP/CHF, community-acquired pneumonia/congestive heart failure; COPD, chronic obstructive pulmonary disease; CVAs, cerebrovascular accidents; F, female; M, male.

### Rectal and naris swabs are consistently obtainable, well tolerated by ICU patients, and suitable for 16S sequencing.

We first assessed the adequacy of each sample type for examining the microbiota of ICU patients based on four factors: ability to obtain samples at prespecified time points, patient tolerability of sampling, density of DNA recovered from the sample, and 16S sequencing suitability. A total of 55 rectal swabs, 57 naris swabs, 57 antecubital swabs, and 15 stool samples (excluding rectal bag samples) were collected during the study period. We successfully collected naris and antecubital swabs in 98% of all attempts and rectal swabs in 97%, with withdrawal of care being the only reason for missed swabs. However, stool was collected in only 31% of attempts, with efforts to collect stool limited in all cases by frequency of bowel movements or placement of a rectal tube (Table 2). Unlike stool, which could be collected only if available, swabs were collected three times a day. Rectal swabs were classified as visibly “soiled” or “unsoiled” by stool. The proportion of samples visibly soiled by stool varied for each patient and between patients over time, with a median of 57% soiled swabs (range, 14% to 100%) (Table 3).

**TABLE 2:** Summary table for percent collection of specimens, detectable DNA recovery, and Good's coverage during sequencing

Specimen type	Collection success rate (%)^a^	Detectable DNA recovery (%)	Median Good's coverage (range)
Rectal swabs	97	99	0.98 (0.96–0.99)^b^
Naris swabs	98	100	1.00 (0.99–1.00)^c^
Antecubital swabs	98	42	0.99 (0.98–1.00)^d^
Stool	31	100	0.99 (0.97–1.00)^b^

a Rectal swab collection success was based on collection of at least 1 swab.

b Sequencing depth of 1,995.

c Sequencing depth of 13,080.

d Sequencing depth of 17,231.

**TABLE 3:** Percentages of soiled versus unsoiled rectal swabs with stool per subject

Subject no.	% of swabs	
	Soiled	Unsoiled
1	43	57
2	83	17
3	57	43
4	67	33
5	100	0
6	50	50
7	43	57
8	14	86
9	100	0

Participants who were able to respond reported no discomfort during the swabbing procedure for naris and antecubital swabs and negligible to mild discomfort during the swabbing procedure for rectal swabs. One participant (subject 6) withdrew on day 5 of 7 due to the discomfort of the turning needed for collection of rectal swabs.

Rectal swabs, naris swabs, and stool met our criterion for adequate DNA density for sequencing (99%, 100%, and 100%, respectively), whereas antecubital swabs had sufficient DNA recovery in only 42% of all swabs collected. All 4 sample types with DNA above our sequencing adequacy threshold were sequenced to an adequate depth for estimation of community composition and diversity (median Good’s coverage of ≥0.98) (Table 2).

### Microbiota from different sample types are compositionally distinct.

**FIG 1:**
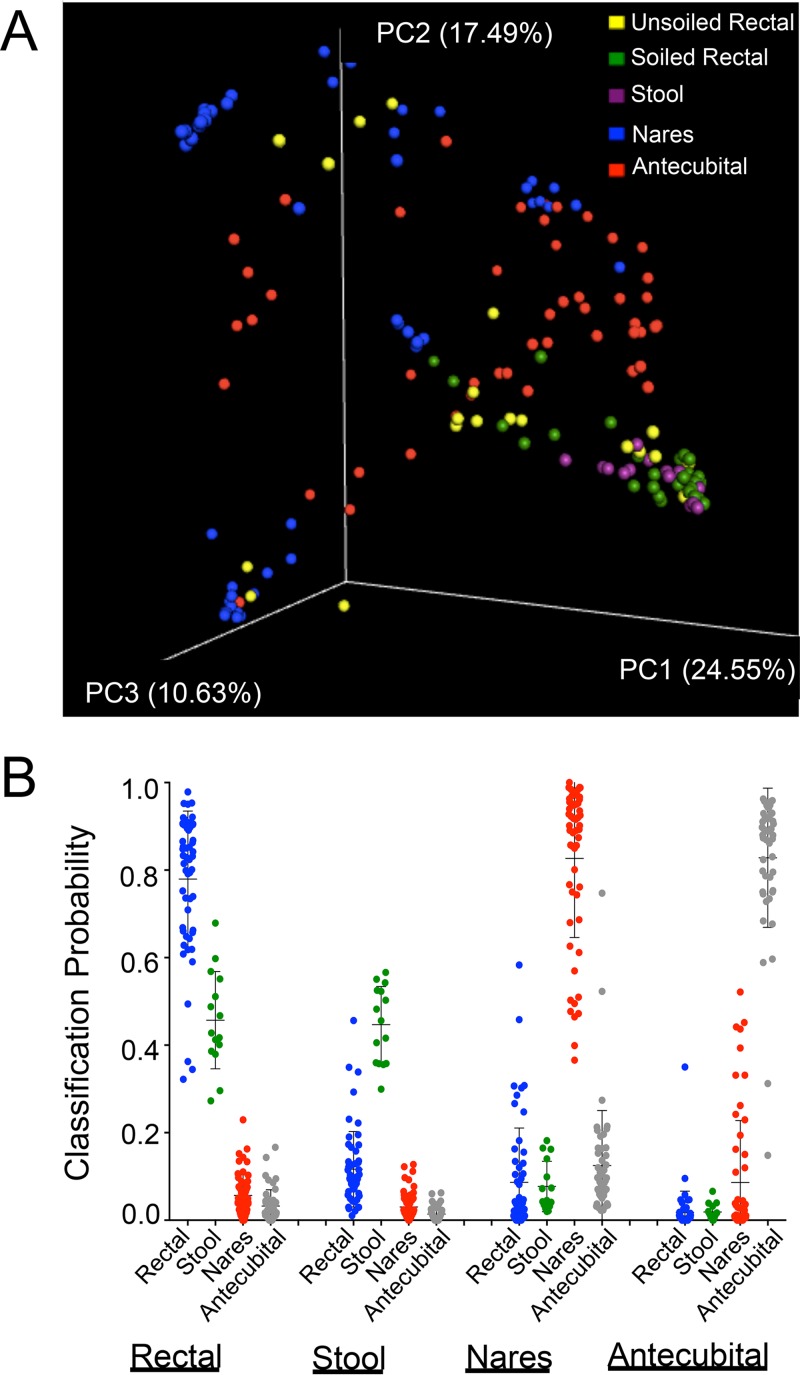
(A) Bray-Curtis principal-coordinate analysis (PCoA) of naris, antecubital, and rectal swabs and stool samples from ICU patients. Bray-Curtis intersample distances were calculated using relative abundance data from taxa representing more than 0.1% of the total population. Axis percentages indicate proportions of the overall variance explained. (B) Classification probabilities (*y* axis) were generated using the script supervised_learning.py (available on QIIME version 1.9.1) and the microbiota composition of each sample type (shown underlined below the *x* axis) as the predictor of the assignment. A high classification probability indicates compositional similarity between the sample and the specified sample type. Data points represent individual samples with means (horizontal line) and standard deviations (error bars).

We compared bacterial community compositions present in the four sample types by 16S rRNA gene sequencing using both phylogenetic methods (weighted UniFrac) and nonphylogenetic methods (Bray-Curtis dissimilarity) with similar results (see Fig. S1 in the supplemental material). Compositional dissimilarity between sample types quantified using Bray-Curtis intersample distances was visualized by principal-coordinate analysis (PCoA) (Fig. 1). Samples were distinguishable based on anatomical site, with rectal swabs and stool providing largely overlapping distributions of community composition (Fig. 1A). Notably, unsoiled rectal swabs appeared compositionally distinct from stool and rectal swabs visibly soiled with stool. We performed a pairwise comparison of levels of beta-diversity between the sample types using permutational multivariate analysis of variance (PERMANOVA) and analysis of similarity (ANOSIM) (see Text S1 in the supplemental material). ANOSIM showed an insignificant difference between rectal swabs and stool (Bray-Curtis *P* value, 0.69; UniFrac *P* value, 0.86), while PERMANOVA showed a significant difference (Bray-Curtis *P* value, 0.002; UniFrac *P* value, 0.019). This difference, although statistically significant, is small (Bray-Curtis pseudo-*F* value = 2.8 and *R* value = −0.03; UniFrac pseudo-*F* value = 3.3 and *R* value = −0.08), indicating a weak effect of any factors distinguishing between them (see Table S1 in the supplemental material). Bacterial composition could be used to assign site of acquisition with a mean classification probability for all three swab types above 0.75 (Fig. 1B), with the most common misclassifications being between rectal swabs and stool.

10.1128/mSphere.00219-18.1 TEXT S1  Supplemental methods describing the use of samples from a published data set (from the study by McDonald et al. [4]), the American Gut Project, and an external validation cohort for comparison with samples from the present study. Download TEXT S1, PDF file, 0.1 MB. Copyright © 2018 Bansal et al. 2018 Bansal et al. This content is distributed under the terms of the Creative Commons Attribution 4.0 International license.

10.1128/mSphere.00219-18.2 FIG S1  Comparison of weighted UniFrac (*y* axis) and Bray-Curtis dissimilarity matrices of antecubital, naris, rectal, and stool samples from 9 critically ill patients by two-sided Mantel test. Spearman’s correlation (ρ) = 0.68, *P* = 0.001. The dissimilarity matrices were generated using the microbiota composition in QIIME2. Download FIG S1, PDF file, 0.2 MB. Copyright © 2018 Bansal et al. 2018 Bansal et al. This content is distributed under the terms of the Creative Commons Attribution 4.0 International license.

10.1128/mSphere.00219-18.9 TABLE S1  Pairwise comparison of levels of beta-diversity in different sample types determined using PERMANOVA and ANOSIM (number of permutations, 999). Bray-Curtis and UniFrac dissimilarities were generated using QIIME2. Download TABLE S1, PDF file, 0.05 MB. Copyright © 2018 Bansal et al. 2018 Bansal et al. This content is distributed under the terms of the Creative Commons Attribution 4.0 International license.

We observed a higher level of community diversity (Shannon diversity index [SDI]) in rectal swabs, antecubital swabs, and stool (median, 4.0, 4.4, and 4.3, respectively) than in naris swabs (1.6). The highest median number of taxa, 48, was detected in antecubital swabs, and the lowest, 9, in naris swabs. A variety of dominant taxa were identified in the different sample types, including taxa from each major phylum (Table 4).

**TABLE 4:** Summary table of median number of genera detected, SDI mean, and most abundant OTUs in each sample type found by 16S rRNA gene sequencing analysis^a^

Sample type	Median no. (range) of genera detected^b^	SDI median (range)	Most dominant OTUs^c^
Rectal	30 (8–44)	4.0 (0.8–5.7)	*Alistipes* (B), *Anaerococcus* (F), *Bacteroides* (B), Clostridium sensu stricto (F), *Corynebacterium* (A), f-*Enterobacteriaceae* (P), f-*Lactobacillaceae* (F), o-*Clostridiales* (F), o-*Lactobacillales* (F), *Prevotella* (B), *Pseudomonas* (P)
Stool	24 (14–32)	4.3 (2.1–5.1)	*Alistipes* (B), *Bacteroides* (B), f-*Enterobacteriaceae* (P), f-*Ruminococcaceae* (F), *Methanobrevibacter* (Arc), o-*Clostridiales* (F), *Parabacteroides* (B)
Naris	9 (3–46)	1.6 (0.2–4.8)	*Corynebacterium* (A), f-*Enterobacteriaceae* (P), f-*Lactobacillaceae* (F), *Prevotella* (B), *Pseudomonas* (P), *Staphylococcus* (F)
Antecubital	48 (8–93)	4.4 (0.6–6.5)	*Bacillus* (F), *Bacteroides* (B), Clostridium sensu stricto (F), *Corynebacterium* (A), f-*Enterobacteriaceae* (P), f-*Lactobacillaceae* (F), *Finegoldia* (F), *Pseudomonas* (P), *Staphylococcus* (F)

a o-, order; f-, family; (Arc), *Archaea*; (F), *Firmicutes*; (B), *Bacteroidetes*; (A), *Actinobacteria*; (P), *Proteobacteria*.

b Specimen RA, >0.1%.

c OTU is most abundant in at least one sample.

### Rectal swabs capture microbial perturbations similar to those in stool samples from ICU patients.

**FIG 2:**
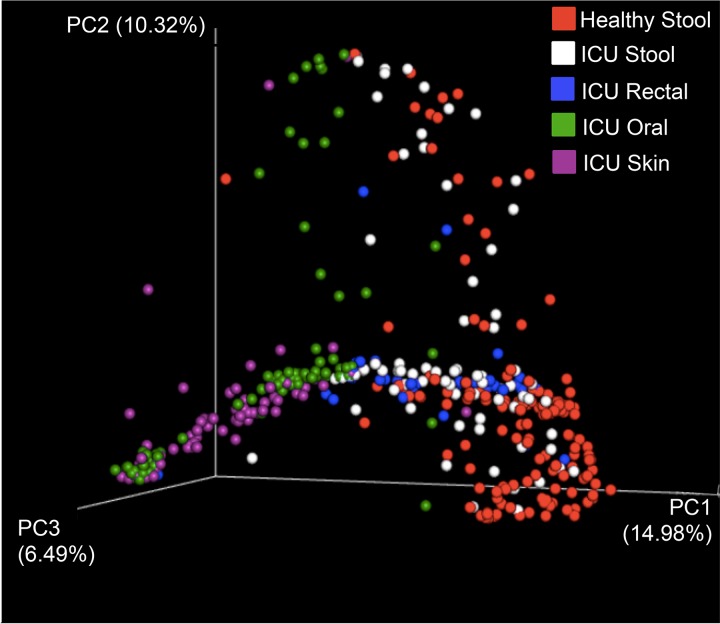
Bray-Curtis principal-coordinate analysis (PCoA) of healthy stool (American Gut Project [AGP] study no. PRJEB11425), stool from ICU patients (from the study by McDonald et al. [4] and the present study), rectal swabs from ICU patients (from the present study), oral and skin samples from ICU patients (from the study by McDonald et al. [4]). Axis percentages indicate proportions of the overall variance explained.

Published comparisons have revealed differences in gut microbial community composition between stool from ICU patients and stool from healthy controls. In order to assess the use of rectal swabs to detect these differences, we compared the first daily swab from our cohort and 27 additional swabs collected from critically ill patients in an unpublished study investigating the effect of a 7-day course of antibiotics versus a 14-day course in comparison to published data from healthy controls and to published 16S profiles from ICU patients (4) and healthy controls from the American Gut Project (AGP) (Text S1). Bray-Curtis intersample distances were visualized by principal-coordinate analysis (Fig. 2). Rectal swabs clustered with stool samples from ICU patients, with a weak effect distinguishing between the two sample types (ANOSIM *R* value = 0.19, *P* = 0.001) (Table 5). No beta-diversity difference was observed between the stool and soiled rectal samples from this study and the ICU stool samples from the study performed by McDonald et al. (Table 5).

**TABLE 5:** Summary table of beta-diversity, alpha-diversity, and taxonomic comparisons between rectal swabs and stool from this study and published data sets

Group 1	Group 2	*R* value (*P* value)
Rectal swabs^a^	ICU stool^b^	0.19 (0.001)
	Healthy stool^c^	0.54 (0.001)
	ICU oral^d^	0.72 (0.001)
	ICU skin^d^	0.80 (0.001)
		
Soiled rectal swabs^e^	ICU stool^b^	−0.04 (0.880)
	Healthy stool^c^	0.38 (0.001)
		
Unsoiled rectal swabs^e^	ICU stool^b^	0.20 (0.001)
	Healthy stool^c^	0.67 (0.001)

a Rectal swabs from present study and external validation cohort.

b Samples from present study and the study by McDonald et al. (4).

c Samples from American Gut Project.

d Samples from McDonald et al. (4) only.

e Samples from present study only.

**FIG 3:**
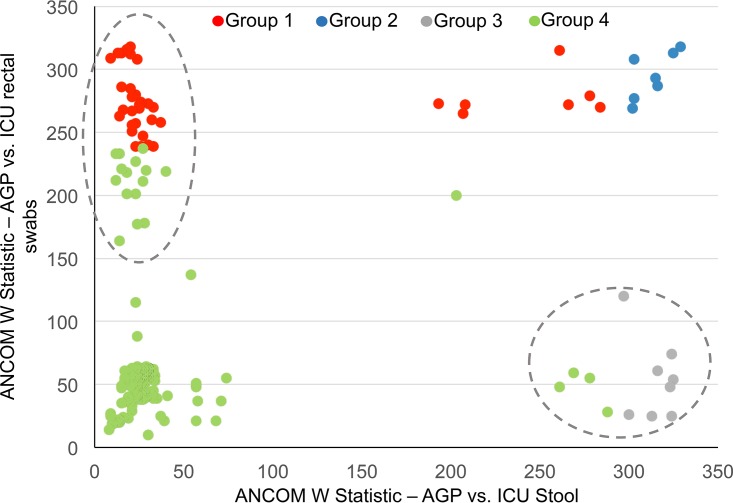
Comparison of taxonomic differences between stool microbiota from healthy patients and stool microbiota from ICU patients and stool from healthy patients and rectal swabs from ICU patients. ANCOM W-statistics were generated for taxa shared between three data sets. The *x* axis presents results of comparisons of stool from healthy volunteers in the American Gut Project (AGP) and stool samples from ICU patients (from the study by McDonald et al. [4] and the present study). The *y* axis presents results of comparisons of stool from the same healthy volunteers with rectal swabs from ICU patients (from the present study). Groups 1 and 3 represent taxa that were discordant between these two comparisons. Taxa that were below the significance level in ANCOM but were still seen to cluster with groups 1 and 3 were included at an arbitrary W statistic value of above 150 and are indicated in the dotted circles. Taxa in these dotted circles form the basis of analysis in Fig. 4 (see also Fig. S2 and S3).

**FIG 4:**
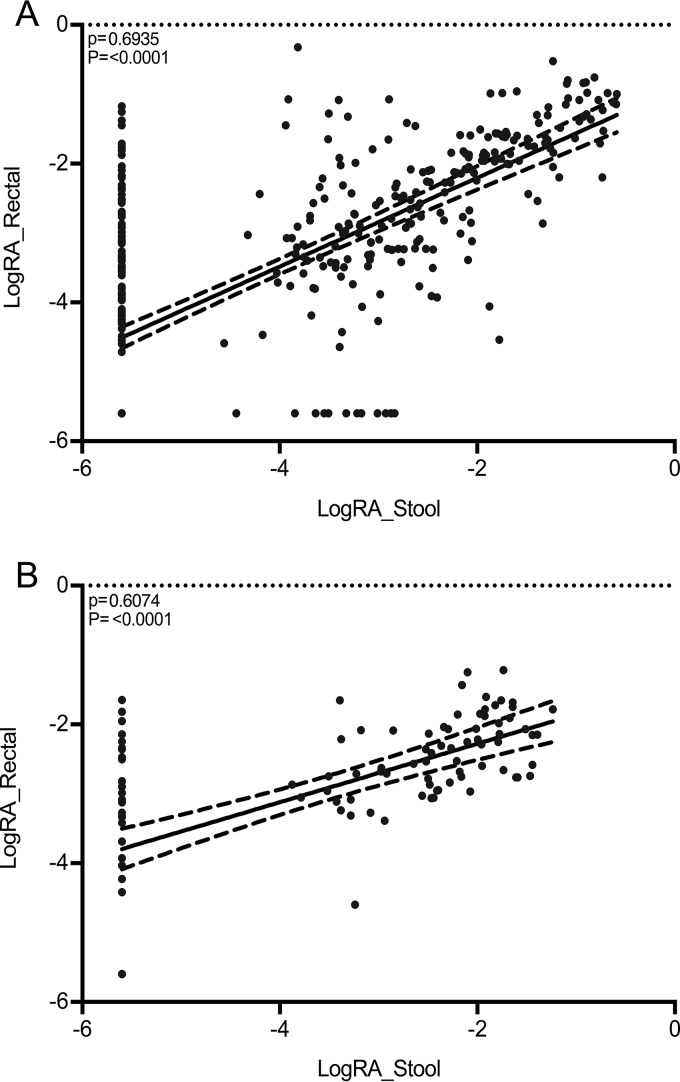
Correlations between logarithmic relative abundance data in paired rectal swabs and stool samples from the present study (matched by patient and day and time of collection) for “discordant” taxa from Fig. 3. (A) Taxa differentially abundant only between stool from healthy volunteers and stool from ICU patients (group 1 in Fig. 3). (B) Taxa differentially abundant only between rectal swabs from ICU patients and stool from healthy volunteers (group 3 in Fig. 3). The Spearman’s correlation coefficient (ρ) value and *P* value for significant results are shown (****, *P* < 0.0001).

We assessed whether taxonomic differences between stool from ICU patients and healthy controls were also observed with rectal swabs. To do so, we compared W statistics determined by ANCOM (analysis of composition of microbiomes) for all taxa present in both data sets for each comparison (ICU swabs versus healthy controls and ICU stool versus healthy controls; Fig. 3). Four groups of taxa (arbitrarily labeled groups 1 to 4 in Fig. 3) were identified as follows: group 1 taxa were differentially abundant in comparisons of rectal swabs from ICU patients and stool from healthy controls but not stool from ICU patients versus healthy controls; group 2 differed in both comparisons; group 3 differed in stool from ICU patients versus healthy controls but not in rectal swabs from ICU patients versus healthy controls; and group 4 differed in neither group. To determine whether “discordance” between these comparisons was more likely due to population or to sample-type-specific factors, we compared the relative abundances (RA) of discordant taxa representing at least 1,000 sequences (in the entire data set) in paired stool/rectal swab samples collected from the same patient on the same day in our primary cohort (Fig. 4; see also Fig. S2 and S3). Taxa that were below the significance level in ANCOM but were still seen to cluster with groups 1 and 3 (circled in Fig. 3) were included in the paired comparisons at an arbitrary W statistic value of above 150 to thoroughly explore differences in any discordant taxa.

10.1128/mSphere.00219-18.3 FIG S2  Correlations between logarithmic relative abundance datain rectal samples and stool samples from the present study matched by patient and day and time of collection for individual taxa differentially abundant only between rectal swabs from ICU patients and stool from healthy volunteers (group 1 in Fig. 3; for overall correlation data, see Fig. 4A). Trendlines for all taxa from Fig. 4A are shown for reference. Download FIG S2, PDF file, 0.2 MB. Copyright © 2018 Bansal et al. 2018 Bansal et al. This content is distributed under the terms of the Creative Commons Attribution 4.0 International license.

10.1128/mSphere.00219-18.4 FIG S3  Correlations between logarithmic relative abundance datain rectal samples and stool samples from the present study matched by patient and day and time of collection for individual taxa differentially abundant only between stool from ICU patients and stool from healthy volunteers (group 3 in Fig. 3; for overall correlation data, see Fig. 4B). Trendlines for all taxa from Fig. 4B are shown for reference. Download FIG S3, PDF file, 0.1 MB. Copyright © 2018 Bansal et al. 2018 Bansal et al. This content is distributed under the terms of the Creative Commons Attribution 4.0 International license.

Although overall agreement for all taxa combined was good for both groups (Spearman correlation coefficients of 0.6935 and 0.6074 for groups 1 and 3, respectively; *P* < 0.0001), several taxa were identified only in rectal swabs, albeit at low relative abundance (Fig. 4). *Faecalibacterium*, *Clostridium*, *Sutterella*, *Pyramidobacter*, *Campylobacter*, *Veillonella*, *Dialister*, *Peptoniphilus*, *Porphyromonas*, *Bifidobacterium*, and *Varibaculum* species were more likely to be detected in rectal swabs than in stool but were generally present at a relative abundance of <0.01 (Fig. S2 and S3).

### Naris and rectal swabs capture interindividual differences in community composition.

**FIG 5:**
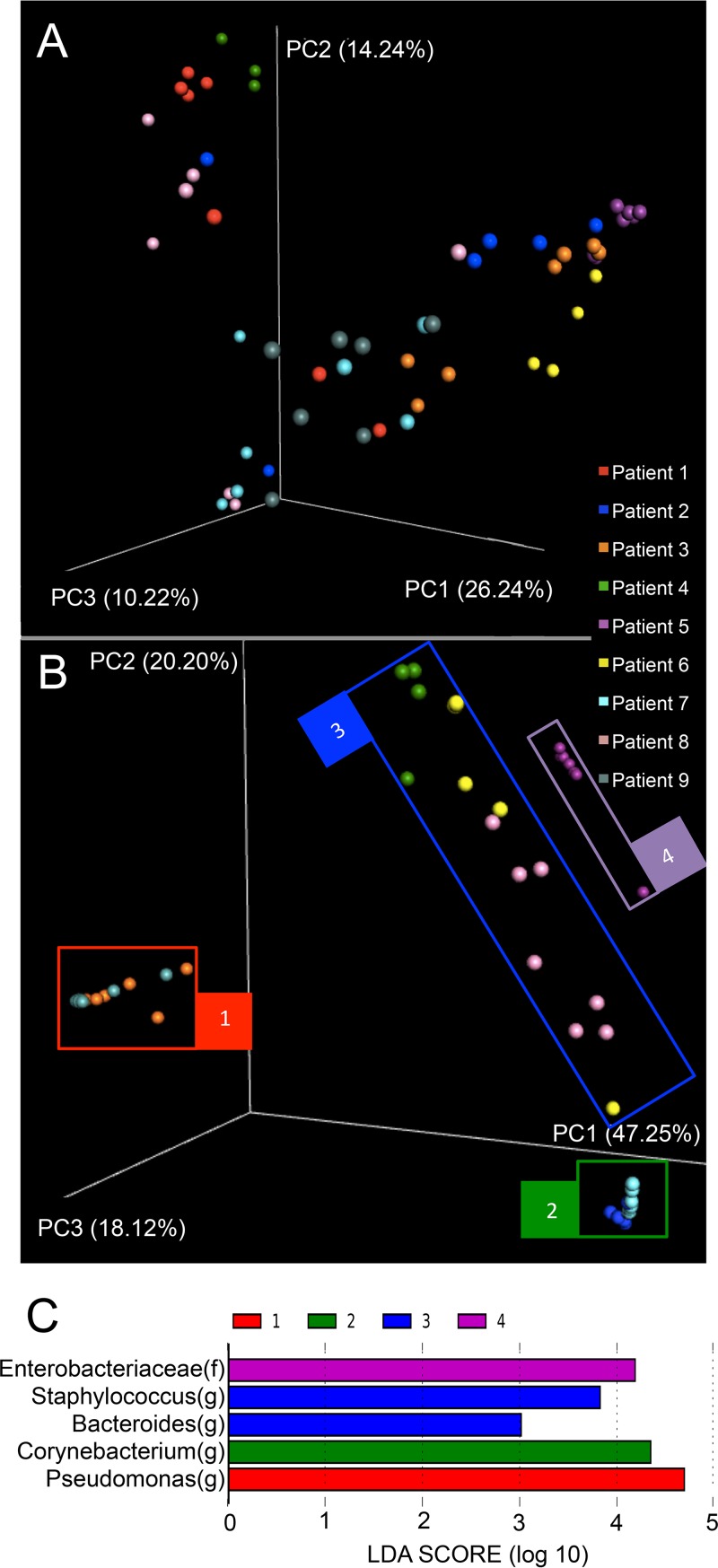
PCoA of Bray-Curtis dissimilarity of sequential daily samples from 9 patients. (A) Rectal swabs. (B) Naris swabs. The colored boxes labeled 1 to 4 represent the sample groups used for LEfSe analysis shown in Fig. 2C. Markers represent sequential daily samples colored according study subject. Bray-Curtis intersample distances were calculated using relative abundance data from taxa representing more than 0.1% of the total population. Axis percentages indicate proportions of the overall variance explained. (C) Taxa identified through LEfSe analysis as primary drivers of differences in microbiota composition between groups 1 to 4 shown in Fig. 5B. f, family; g, genus; LDA, linear discriminant analysis.

Since only rectal and naris swabs were easily obtained and consistently suitable for sequencing, we assessed their ability to resolve interindividual differences in community composition. Both swab types demonstrated clear interindividual variability in microbial community composition. Compositional clustering was evident in naris samples, whereas more overlap in beta-diversity was evident in rectal swabs (Fig. 5A and B). Clustering of communities from the nares was driven by a relatively small number of taxa. Linear discriminant analysis effect size (LEfSe) analysis of clusters identified by PCoA of naris swabs determined that *Enterobacteriaceae*, *Staphylococcus*, *Bacteroides*, *Corynebacterium*, and *Pseudomonas* were the primary drivers of between-cluster differences (Fig. 5C).

### The rectal microbiota demonstrates greater daily variation than the naris microbiota.

We assessed whether either swab type could resolve within-patient temporal changes in microbial community structure by assessing the daily variability and trends in alpha-diversity as well as the degree of Bray-Curtis dissimilarity in samples obtained on sequential days.

**FIG 6:**
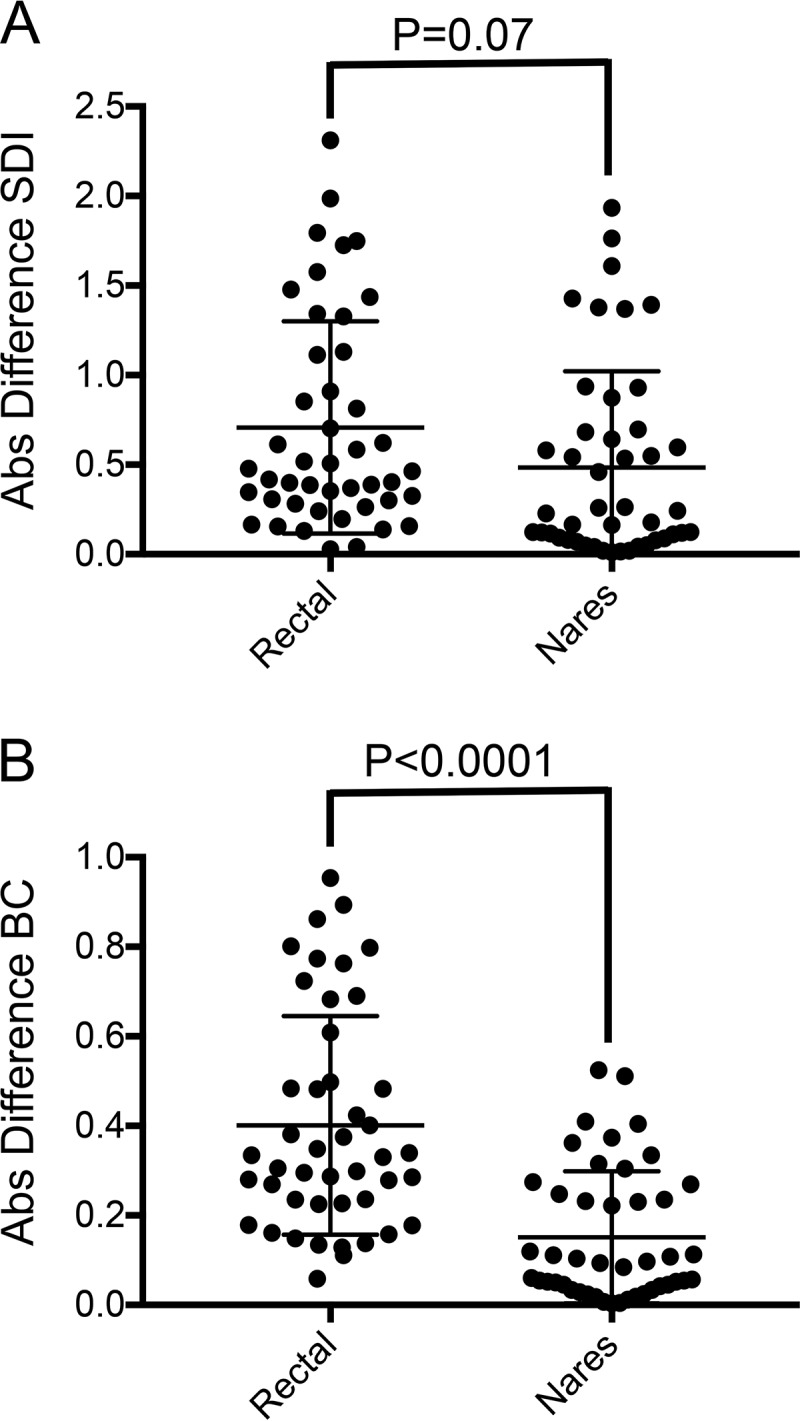
Absolute (Abs) differences in Shannon diversity index (SDI) values (A) and Bray-Curtis (BC) dissimilarity index values (B) between sequential daily rectal and naris samples in each patient. Data points represent sequential sample comparisons with means and standard deviations (solid horizontal line and error bars). *P* values for pairwise Wilcoxon matched-pair signed-rank tests are shown.

Daily differences in Bray-Curtis dissimilarity matched by day and patient were significantly greater for rectal swabs than for naris swabs (Fig. 6). Paired daily differences in alpha-diversity were also greater for rectal swabs than for naris swabs (*P* = 0.07). Several study subjects (subjects 6, 7, and 8) showed a progressive decline in rectal swab alpha-diversity over the study period without a corresponding decline in naris diversity (Fig. S4).

10.1128/mSphere.00219-18.5 FIG S4  Daily variations in Shannon diversity index (SDI) values for rectal (green line), naris (red line), and antecubital (blue line) microbiota of 9 ICU patients over a 7-day sampling period. Download FIG S4, PDF file, 0.1 MB. Copyright © 2018 Bansal et al. 2018 Bansal et al. This content is distributed under the terms of the Creative Commons Attribution 4.0 International license.

### Rectal swabs, but not nasal swabs, correlate with plasma inflammatory markers.

**FIG 7:**
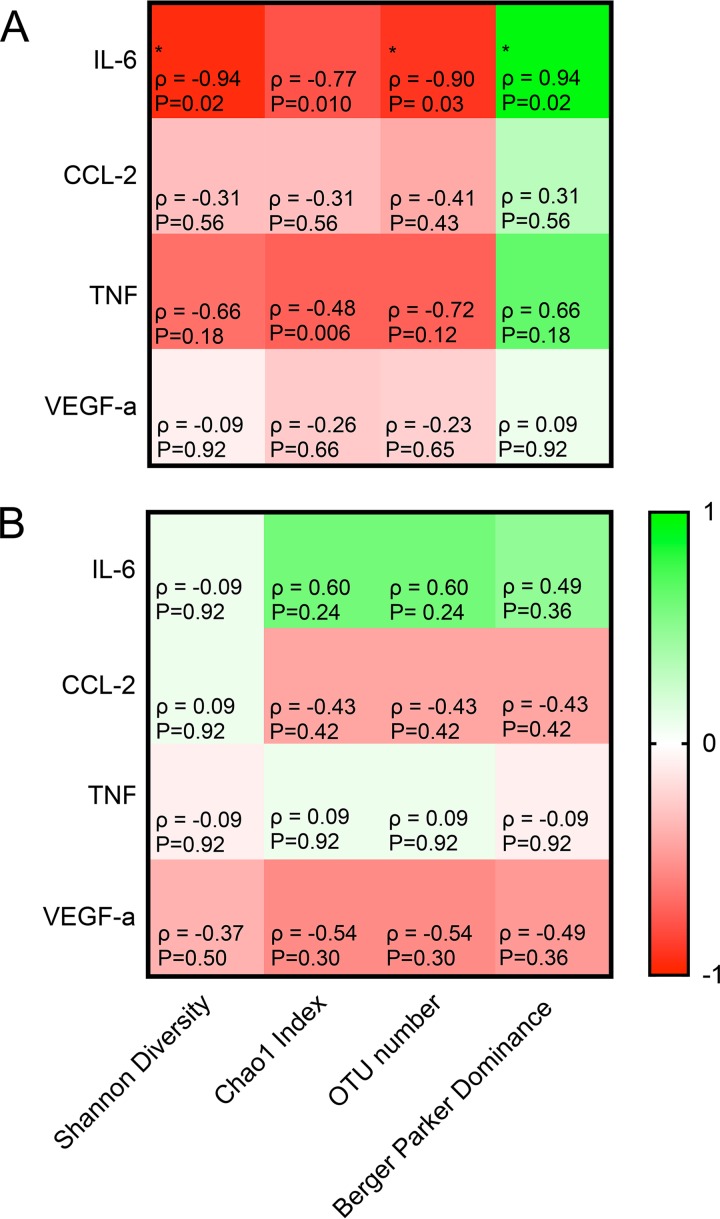
Heat maps summarizing Spearman correlations between log_10_-transformed cytokine levels (log IL-6, CCL-2, TNF, and VEGF-A [in picograms/milliliter]) in blood plasma and composite measures of alpha-diversity (Shannon diversity index, Chao1 index, OTU number, Berger-Parker dominance value) in day 1 rectal (A) and naris (B) swabs from 6 study subjects for whom blood samples were available. Green indicates a positive correlation, and red indicates a negative correlation. The shade of the color represents the strength of the correlation, with lighter shades indicating a weaker correlation. The Spearman’s correlation coefficient (ρ) value and *P* value for significant results are shown (*, *P* < 0.05; **, *P* < 0.01; ***, *P* < 0.001; ****, *P* < 0.0001).

Three patients (subjects 4, 6, and 8) who had persistently low rectal mucosal microbiota diversity (Shannon diversity index, <2) or had a shift toward low diversity during the study period died within 28 days (Fig. S4). However, since the size of our study was insufficient to power microbiota-outcome correlations, we sought to compare microbial community composition to the more proximal measure of patient status of circulating inflammatory markers. We assayed plasma levels of interleukin-6 (IL-6), chemokine (C-C motif) ligand-2 (CCL-2), tumor necrosis factor (TNF), and vascular endothelial growth factor-A (VEGF-A) and correlated these levels with measures of alpha-diversity derived from 16S sequencing for each patient on the day of enrollment. Shannon diversity index (SDI) values (representing a composite of community evenness and richness), Chao1 values (measuring levels of singletons or rare taxa in the community), observed operational taxonomic unit (OTU) numbers, and Berger-Parker dominance values (indicating the dominance of a single taxon in a community) were calculated and compared to cytokine levels. Negative correlations between alpha-diversity and 3 of 4 inflammatory cytokines were observed for rectal swab microbiota, but correlations that were only inconsistent or weak for two cytokines were noted for nasal swabs (Fig. 7). These correlations were largely driven by between-patient differences at both the community diversity and cytokine levels (Fig. S5 and S6).

10.1128/mSphere.00219-18.6 FIG S5  Comparisons of log_10_-transformed cytokine (log IL-6, CCL-2, TNF, and VEGF-A) levels (in picograms per milliliter) in blood plasma and alpha-diversity indices (Shannon diversity index, Chao1 index, OTU number, Berger-Parker dominance value) in rectal swabs from 6 study subjects. Marker color represents samples from the same patient in each panel of this figure. Download FIG S5, PDF file, 0.1 MB. Copyright © 2018 Bansal et al. 2018 Bansal et al. This content is distributed under the terms of the Creative Commons Attribution 4.0 International license.

10.1128/mSphere.00219-18.7 FIG S6  Comparisons of log_10_-transformed cytokine (log IL-6, CCL-2, TNF, and VEGF-A) levels (in picograms per milliliter) in blood plasma and alpha-diversity indices (Shannon diversity index, Chao1 index, OTU number, Berger-Parker dominance value) in naris swabs from 6 study subjects. Marker color represents samples from the same patient in each panel of this figure. Download FIG S6, PDF file, 0.1 MB. Copyright © 2018 Bansal et al. 2018 Bansal et al. This content is distributed under the terms of the Creative Commons Attribution 4.0 International license.

### The Gram composition of 16S sequencing data differs from Gram staining results in stool samples of ICU patients.

Since Gram staining has been proposed as a “rapid” tool for the assessment of low-diversity communities in cases of critical illness (17, 18), we assigned Gram-positive (Gram^+^) and Gram^−^ labels to all taxa (relative abundance [RA], >1%) present in 16S sequencing data of the 15 patient stool samples and compared the total 16S Gram^+^ and Gram^−^ proportions in a sample to the proportions determined by Gram staining. Gram staining data from stool samples and 16S data agreed (within 10%) for a single patient, patient 2, but differed by more than 20% in half of the samples assessed, suggesting potentially poor agreement between these two methods (Table 6; see also Fig. S7).

10.1128/mSphere.00219-18.8 FIG S7  (A) Percentage of Gram-positive and Gram-negative bacteria in stool samples as determined by Gram staining and ImageJ analysis. (B) Microbial composition of the stool samples as determined by 16S rRNA gene sequencing, with taxa classified as Gram positive colored in shades of blue and taxa classified as Gram negative colored in shades of red. The BacMap Genome Atlas and the Integrate Microbial Genomes (IMG) database were used to classify taxa identified by 16S sequencing (RA, >1%) as Gram positive or Gram negative. Download FIG S7, PDF file, 0.2 MB. Copyright © 2018 Bansal et al. 2018 Bansal et al. This content is distributed under the terms of the Creative Commons Attribution 4.0 International license.

**TABLE 6:** Summary table for differences in total proportions of Gram^+^ and Gram^−^ results assigned to 16S data and as determined by Gram staining for stool samples from ICU patients

Subject no.	% difference (16S [Gram^+^] – Gram staining [Gram^+^])	% difference (16S [Gram^−^] – Gram staining [Gram^−^])
2	7.6	−8.4
2	5.5	−7.7
3	14.6	−15.3
3	16.0	−16.7
3	15.7	−16.3
5	−8.9	8.8
5	49.9	−51.4
5	56.0	−56.3
7	21.9	−23.2
7	29.9	−32.9
9	45.0	−47.1
9	64.5	−67.0
9	50.7	−52.2
9	39.1	−40.9
9	52.5	−54.3

## DISCUSSION

The primary aim of this study was to identify sample types that describe potentially informative within- and between-patient differences in microbiota composition in a generalized ICU population. We proposed the following criteria for utility: (i) they must be obtainable from patients when needed for study purposes; (ii) they must have sufficient microbial DNA to successfully assay community composition; (iii) they should resolve within-patient (temporal) and between-patient differences in community composition; and (iv) they should successfully capture putative disease-dysbiosis relationships. In our study, only rectal swabs met all four criteria. Stool samples were frequently not obtainable, due to either decreased bowel movement frequency or the presence of a rectal tube, both of which represent common impediments to stool collection in ICU patients. There was low DNA recovery from antecubital swabs, likely due to the use of daily chlorhexidine bathing, which substantially reduces bacterial colonization (19). Swabs of the nares were readily obtainable, suitable for sequencing, and compositionally distinct from rectal microbiota. However, unlike the rectal swabs, they failed to capture the daily community diversity and composition variation as well as diversity-inflammation correlations observed with rectal swabs.

Importantly, even in this small cohort designed to assess technical utility, we observed important microbiota-disease-treatment relationships using rectal swabs consistent with prior published reports describing the microbiota in critical illness. The compositional shift of a healthy microbiome toward a low-diversity state dominated by a single taxon during critical illness has been well described and linked to immune dysregulation and clinical outcomes (10–16, 20–23). In this study, rectal swabs successfully captured perturbations of the microbiota similar to those that have been previously described using stool from critically ill patients. We observed ecological collapse and emergence of a dominant pathogenic genus during antibiotic treatment for nosocomial sepsis in one study subject, loss of microbially diverse populations during a short sampling interval in a subset of 3 subjects who died within 28 days, and an inverse correlation between taxonomic diversity and markers of systemic inflammation. These findings recapitulate several disease-microbiota relationships in other critical care studies (4, 24), supporting the use of this sample type for future studies.

There are several important caveats and limitations with regard to this study. The small sample size precludes any formal assessment of the sensitivity and specificity of rectal swabs for detection of disease or outcome-associated patterns of community composition, since all types of community composition were not captured. Changes in absolute bacterial burden within or between patients could not be measured, as the quantity of biological specimen can vary greatly between swabs. In 5 patients analyzed, there was poor agreement between the Gram staining results and the 16S sequencing-assigned Gram characteristics. Although our study was underpowered with respect to drawing any conclusions from this finding, we feel that this technique requires prospective validation and optimization before being applied as a metric of community composition (17, 18).

Importantly, we found that the presence of visible stool on rectal swabs affected community composition. There are conflicting reports in the literature about the extent of contamination by skin bacteria from the anal verge in rectal swabs (25, 26). Our findings indicate the importance of either standardization of this swab characteristic for observational studies or incorporation of this variable as a confounder of community structure into data analyses. An ideal study design might restrict inclusion to only soiled or unsoiled swabs; however, a pragmatic design might include both types but restrict analyses to features that may be consistently identified in both sample types (for example, dominance by a single pathogenic taxon). Indiscriminate exclusion of unsoiled swabs might unnecessarily discard potentially informative samples. For example, using unsoiled swabs, we observed a large-scale shift in diversity in one study subject (subject 8) that was potentially biologically informative but that would not have been observed if only visibly soiled swabs had been processed.

Community composition and temporal changes differed between naris and rectal swabs within patients, indicating that these two sample types represent compositionally distinct communities that may provide complementary information. While rectal swabs demonstrated stronger correlations with circulating cytokines, this might not be true in all cases or under all clinical conditions. For example, perturbation of naris microbiota has been previously correlated with specific infections (27). Given the ease with which the naris swabs were obtained, they represent an additional target for sampling for the detection of unique associations with disease outcomes and treatment responsiveness during critical illness.

In conclusion, rectal and naris swabs are easy to acquire, are suitable for 16S rRNA gene sequencing, and can be used to describe within- and between-subject differences in microbiota of critically ill patients and thus represent a practical alternative to stool samples for use in studies of the microbiota in critical illness.

## MATERIALS AND METHODS

### Study design and patient enrollment.

This was a prospective observational study designed to assess the utility of rectal, naris, and antecubital swabs and stool in analyzing the microbiome of critically ill patients. Consenting patients were enrolled in a 27-bed medical-surgical ICU at Lakeridge Health Oshawa (LHO), a community hospital in Ontario, Canada. A convenience sample of patients consecutively admitted over a 2-week period from whom consent to participate was obtained were included in the study. Adult patients who had not undergone a colostomy were considered eligible patients. Patient enrollment and specimen collection took place at LHO, while sample processing and analysis were carried out at University Health Network (UHN).

This study was approved by the institutional Research Ethics Boards at LHO and UHN.

### Samples and data collection.

Daily rectal-mucosal, anterior naris, and antecubital-fossa swabs were collected from patients for a period of 7 consecutive days (or as long as they stayed in the ICU) from the day of consent. In accordance with Human Microbiome Project (HMP) guidelines and protocols (28), Catch-All sample collection swabs (Epicentre Biotechnologies, Madison, WI) were premoistened with sterile SCF-1 solution (50 mM Tris buffer [pH 7.6], 1 mM EDTA [pH 8.0], and 0.5% Tween 20) and used for collection of samples from all three anatomical sampling sites. Rectal swabs were collected by inserting the swab 1 to 2 cm past the anal verge and gently rubbing the rectal mucosal surface. Naris swabs were collected by rubbing the mucosal surface of the anterior nares, going around the area twice. Antecubital swabs were collected by holding the swab so that the shaft was parallel to the skin surface and rubbing the swab back and forth approximately 50 times along the antecubital crease. After collection, swabs were placed in a sterile empty tube. Stool was collected from patients in a sterile polypropylene container (Starplex Scientific Inc., Etobicoke, Ontario, Canada). Stool samples from rectal bags were excluded for analysis in this study. All samples were stored on ice immediately after collection and transferred to a freezer (−80°C) within 1 h. Samples were stored at −80°C and processed within 4 weeks. Rectal swabs were collected three times a day, but only the first daily sample was used for analysis.

In addition, age, sex, primary diagnosis, clinical status, antimicrobial therapy, leukocyte count, and temperature were recorded over the sampling period for each of the patients.

### DNA extraction and quantitation.

DNA extraction from swabs and stool was performed using a DNeasy PowerSoil kit (Qiagen, Carlsbad, CA) with changes as suggested in the HMP protocol (28). After thawing, approximately 0.25 g of stool and 0.25 g of the swabs were each transferred into PowerBead tubes (Qiagen, Carlsbad, CA) containing 750 µl of specimen collection fluid. Tubes containing the swabs were subjected to vortex mixing for 5 min, and the swabs were wrung out by being pressed against the inner side of the tube before removal to ensure maximal DNA yield. Finally, DNA was eluted in 30 µl of DNA-free PCR-grade water (Qiagen, Carlsbad, CA). No other changes were made to the procedure. The total DNA in each sample was quantified using a Qubit fluorimeter (model 3.0) and a Qubit double-stranded (dsDNA) HS (high-sensitivity) assay kit following the protocol of the manufacturer (Invitrogen, Carlsbad, CA). Any sample with a DNA concentration below the standard curve (quantification range, 0.2 to 100 ng dsDNA) was considered below the limit of quantification for the purpose of this study.

### Sequencing and bioinformatics.

The isolated DNA was sequenced at the Centre for the Analysis of Genome Evolution and Function (CAGEF) at the University of Toronto. The V4 hypervariable region of the 16S rRNA gene was amplified using a universal forward sequencing primer and a uniquely bar-coded reverse sequencing primer to allow multiplexing (29). Primers contained an adapter sequence to bind the amplicons to the Illumina flow cell. PCR-based library construction was performed in triplicate 25-µl solutions containing 1× KAPA2G Robust HotStart ReadyMix, a 600 nM concentration of each primer, and 1 µl of DNA template. For every PCR, sterile distilled water (dH_2_O) was used as a negative control to ensure that no contaminating DNA was present. PCR conditions were 95°C for 3 min; 18 to 33 cycles of 95°C for 15 s, 50°C for 15 s, and 72°C for 15 s; and 72°C for 5 min. All PCRs were run on a 1% agarose Tris-borate-EDTA (TBE) gel to visualize the amplification and to approximate the DNA quantity. Triplicates were pooled and combined using approximately even concentrations based on the gel images to create the final library. The final library was purified using 0.8× volumes of Agencourt AMPure XP beads (Beckman Coulter, Inc., Indianapolis, IN) according to the manufacturer’s protocol and quantified using a Qubit fluorometer. The final library was prepared according to the MiSeq user guide, diluted to a concentration of 7 pM, and combined with a 15% PhiX control. Sequencing was performed using V2 chemistry (150 bp ×2) on an Illumina MiSeq platform (Illumina, San Diego, CA).

The UPARSE pipeline, available through USEARCH, was used for sequence analysis (30, 31). Sequences were assembled and quality trimmed using –fastq_mergepairs and –fastq_filter, with a –fastq_maxee set at 1.0 and 0.5, respectively. Following the use of the UPARSE pipeline, merged pairs were then dereplicated and sorted to remove singletons. Sequences were clustered into operational taxonomic units (OTUs) at 97% identity. Chimeras were detected and removed using the –uchime_ref reference-based method and the Ribosomal Database Project (RDP) “gold” 16S database, derived from the RDP training set version 9 and accessed through USEARCH (32). Assembled sequences were then mapped back to the chimera-free OTUs. Taxonomy assignment was executed using –utax (available through USEARCH). A minimum confidence level of 0.8 was required for taxonomy assignment. OTU sequences were aligned using PyNast, accessed through Quantitative Insights Into Microbial Ecology (QIIME) (33). Diversity indices of samples were rarefied to 1,995 sequences for stool and rectal swabs and to 13,080 sequences for naris swabs to account for the effects of sequencing depth on diversity. Since antecubital samples are low-density samples and prone to contamination, we used taxonomic composition of negative controls to filter for contaminants. After removal of singletons, OTUs with a maximum sequence of less than <0.01% of total sequence abundance for all antecubital samples were removed. Using the resulting table, relative abundance data were calculated and OTUs whose maximum relative abundance from negative controls exceeded the maximum relative abundance in samples (i.e., the ratio of maximum RA_controls_/maximum RA_samples_ = >1) were removed. Antecubital samples were rarefied to 17,231 sequencing reads for calculation of diversity metrics. We applied a relative abundance cutoff value, and OTUs representing less than 0.1% of the total population were discarded for all sample types for all further analysis.

### Cytokine analysis.

Biomarker quantification was performed using a multiplex Ella platform (ProteinSimple, San Jose, CA) (34, 35). Four biomarkers (TNF, IL-6, CCL-2, and VEGF-A) were selected, and assays were done according to the manufacturer’s protocol. Plasma samples were collected for all patients except patients 1 and 3. For two study subjects, patients 2 and 7, only plasma samples from study days 1 to 3 and 7 were included for cytokine analysis as these patients remained stable throughout the study period and it was assumed that daily sampling at the beginning of the study and then on the last study day would capture any significant changes in cytokine levels. Samples for patient 6 were excluded as that patient withdrew from the study on day 5, resulting in an incomplete sample set. Plasma samples were thawed overnight at 4°C and diluted 1:10 using diluents supplied by the manufacturer. A 50-µl volume of the diluted plasma was added to the sample cartridge and then inserted into the Ella instrument, requiring no additional user intervention. Raw data were analyzed using SimplePlex Explorer software.

### Gram staining.

Gram staining was performed on stool samples using strains of Staphylococcus aureus (Gram-positive) and Pseudomonas aeruginosa (Gram-negative) as standards. Catch-All sample collection swabs (EPICENTRE Biotechnologies, Madison, WI) were used to smear samples on glass slides, and stool slides were swabbed with phosphate-buffered saline (PBS) to reduce the bacterial concentration. The protocol of the Gram staining kit manufacturer (Remel, Lenexa, KS) was followed for staining, with no changes. Slides were viewed under an Olympus BX-41 microscope using the 100× oil immersion objective. Images were captured using a Lumenera INFINITY2 camera.

### Bacterial cell counts.

ImageJ (36, 37) software was used to count the number of Gram-positive and Gram-negative cells. Color threshold counting was used to determine the number of cells by adjusting the color threshold and then analyzing the cells of a specific color.

### Classification of 16S data into Gram-positive and Gram-negative data.

A search was done on the BacMap Genome Atlas (38) and the Integrated Microbial Genomes (IMG) database (39) to classify taxa identified by 16S sequencing (RA, >1%) as Gram^+^ or Gram^−^. There were a total of 23 taxa classified as Gram^+^ and 19 taxa classified as Gram^−^. One of the taxa was classified as “Other” as it was identified only by its domain. The total proportion of Gram^+^ or Gram^−^ taxa in a sample was determined by aggregating the relative abundances of each of the Gram^+^ or Gram^−^ taxa present in the sample.

### Statistical analysis.

QIIME was used to generate the PCoA plots of Bray-Curtis intersample distances and the classification probabilities (33). Classification probabilities were generated using the supervised_learning.py script (40) and the microbiota composition of each sample type as the predictor of assignment. LEfSe (linear discriminant analysis effect size) was used to determine features that differentiated the microbial communities of two or more groups (41). The Wilcoxon matched-pair signed-rank test was used to compare nonparametric continuous data. *P* values of <0.05 were considered statistically significant. Spearman’s correlation coefficient was used as a measure of the statistical strength of a monotonic relationship between two variables (Fig. 7). All data analyses were performed and figures created using GraphPad Prism (GraphPad Software, Inc., San Diego, CA).

### Accession number(s).

All 16S rRNA gene sequence data have been deposited into the NCBI Sequence Read Archive under accession numbers PRJNA400445 and SRP116317.

10.1128/mSphere.00219-18.10 DATA SET S1  Strategy used to filter likely contaminants from sequencing data of antecubital samples used in the present study. Download DATA SET S1, XLSX file, 6.6 MB. Copyright © 2018 Bansal et al. 2018 Bansal et al. This content is distributed under the terms of the Creative Commons Attribution 4.0 International license.
