# Erratum for Bansal et al., “Rectal and Naris Swabs: Practical and Informative Samples for Analyzing the Microbiota of Critically Ill Patients”

**DOI:** 10.1128/mSphere.00328-18

**Published:** 2018-06-27

**Authors:** Saumya Bansal, Jenny P. Nguyen, Aleksandra Leligdowicz, Yu Zhang, Kevin C. Kain, Daniel R. Ricciuto, Bryan Coburn

**Affiliations:** a Department of Laboratory Medicine and Pathobiology, University of Toronto, Toronto, ON, Canada; b University of Toronto, Interdepartmental Division of Critical Care, Toronto, ON, Canada; c University Health Network, Division of Critical Care Medicine, Toronto, ON, Canada; d Department of Medicine, University Health Network, Toronto, ON, Canada; e Department of Medicine, Division of Infectious Diseases, University of Toronto, Toronto, ON, Canada; f Lakeridge Health, Division of Infectious Diseases, Oshawa, ON, Canada

## ERRATUM

Volume 3, no. 3, e00219-18, 2018, https://doi.org/10.1128/mSphere.00219-18. Page 5 of the PDF: the percentages in Fig. 2 were incorrectly transcribed. Please see below for the correctly labeled figure.

**FIG 2:**
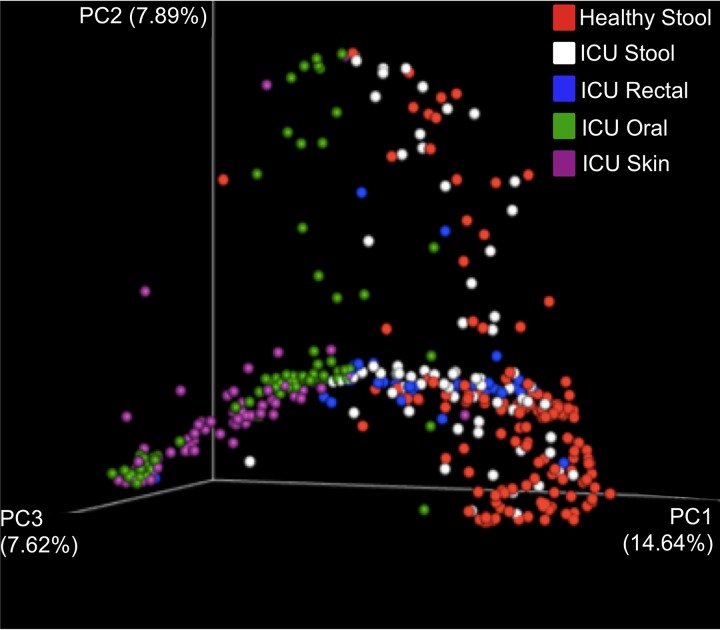
Bray-Curtis principal-coordinate analysis (PCoA) of healthy stool (American Gut Project [AGP] study no. PRJEB11425), stool from ICU patients (from the study by McDonald et al. [4] and the present study), rectal swabs from ICU patients (from the present study), oral and skin samples from ICU patients (from the study by McDonald et al. [4]). Axis percentages indicate proportions of the overall variance explained.

