# Supplementary material for: Rectal and Naris Swabs: Practical and Informative Samples for Analyzing the Microbiota of Critically Ill Patients
Source: mSphere. 2018 Jun 13;3(3):e00219-18. doi: 10.1128/mSphere.00219-18 (PMC6001609; doi:10.1128/mSphere.00219-18)
Supplement: TEXT S1 [file sph003182568s1.pdf]

Text S1: Supplementary Methods

16S rRNA gene sequences from publically available datasets

We obtained 16S rRNA gene sequences for stool, skin, and oral samples from critically ill patients (Study No.: PRJEB11425) enrolled in the McDonald *et al.* study (1) and stool samples from American Gut Project (AGP) healthy volunteers (Study No: PRJEB11419) from the European Nucleotide Archive (ENA) database. We used a single sample from each patient or healthy volunteer in the comparative analysis for a total of 448 samples – 92 ICU oral samples, 86 ICU skin samples, 77 ICU stool sample (from McDonald *et al.* study), 150 AGP healthy stool samples, 27 rectal swab samples from an external cohort of critically ill patients, 9 rectal swab samples and 7 stool samples from patients enrolled in the present study.

Bioinformatics and Sequencing pipeline

Samples from the present study, the external validation cohort, the McDonald *et al.* study, and the American Gut Project were pipelined together using tools available on QIIME 2 - v 2017.XX (2). Sequence data from the four datasets were individually imported as QIIME 2 artifacts and filtered using DADA2 (3). Forward reads truncated at 100 bp from each sample were used to minimize any run specific factors. The denoised and quality filtered data were then merged using the *feature-table* plugin (4). Taxonomy was assigned using trained classifier using the Greengenes database (version 13.8) at 97% sequence identity. For phylogenetic analysis, the *alignment* and *phylogeny* plugins were used to generate a mid-point rooted tree (5).

Statistical Analysis

We used the *diversity* plugin, available through the QIIME 2 tool set, to generate Bray-Curtis dissimilarity matrix, weighted Unifrac dissimilarity matrix (6, 7) and PCoA plots. A two-sided Mantel test was used to identify correlation between dissimilarity matrices. Pairwise comparison of beta-diversity in each of the sample types was done using the non-parametric tests PERMANOVA and

ANOSIM. Bonferroni correction was applied to p-values when multiple comparisons were made. ANCOM was used to test for differentially abundant taxa between groups (8)

References

1. McDonald D, Ackermann G, Khailova L, Baird C, Heyland D, Kozar R, Lemieux M, Derenski K, King J, Vis-Kampen C, Knight R, Wischmeyer PE. 2016. Extreme Dysbiosis of the Microbiome in Critical Illness. mSphere 1:e00199-16.
2. Caporaso JG, Kuczynski J, Stombaugh J, Bittinger K, Bushman FD, Costello EK, Fierer N, Peña AG, Goodrich JK, Gordon JI, Huttley GA, Kelley ST, Knights D, Koenig JE, Ley RE, Lozupone CA, McDonald D, Muegge BD, Pirrung M, Reeder J, Sevinsky JR, Turnbaugh PJ, Walters WA, Widmann J, Yatsunenko T, Zaneveld J, Knight R. 2010. QIIME allows analysis of high-throughput community sequencing data. Nat Methods 7:335–6.
3. Callahan BJ, McMurdie PJ, Rosen MJ, Han AW, Johnson AJA, Holmes SP. 2016. DADA2: High-resolution sample inference from Illumina amplicon data. Nat Methods 13:581–583.
4. McDonald D, Clemente JC, Kuczynski J, Rideout JR, Stombaugh J, Wendel D, Wilke A, Huse S, Hufnagle J, Meyer F, Knight R, Caporaso JG. 2012. The Biological Observation Matrix (BIOM) format or: how I learned to stop worrying and love the ome-ome. Gigascience 1:7.
5. Katoh K, Standley DM. 2013. MAFFT multiple sequence alignment software version 7: improvements in performance and usability. Mol Biol Evol 30:772–80.
6. Lozupone C, Knight R. 2005. UniFrac: a new phylogenetic method for comparing microbial communities. Appl Environ Microbiol 71:8228–35.
7. Lozupone CA, Hamady M, Kelley ST, Knight R. 2007. Quantitative and qualitative beta diversity measures lead to different insights into factors that structure microbial communities. Appl Environ Microbiol 73:1576–85.
8. Mandal S, Van Treuren W, White RA, Eggesbø M, Knight R, Peddada SD. 2015. Analysis of composition of microbiomes: a novel method for studying microbial composition. Microb Ecol Health Dis 26:27663.
