# Supplementary figures and images for: Rectal and Naris Swabs: Practical and Informative Samples for Analyzing the Microbiota of Critically Ill Patients
Source: mSphere. 2018 Jun 13;3(3):e00219-18. doi: 10.1128/mSphere.00219-18 (PMC6001609; doi:10.1128/mSphere.00219-18)

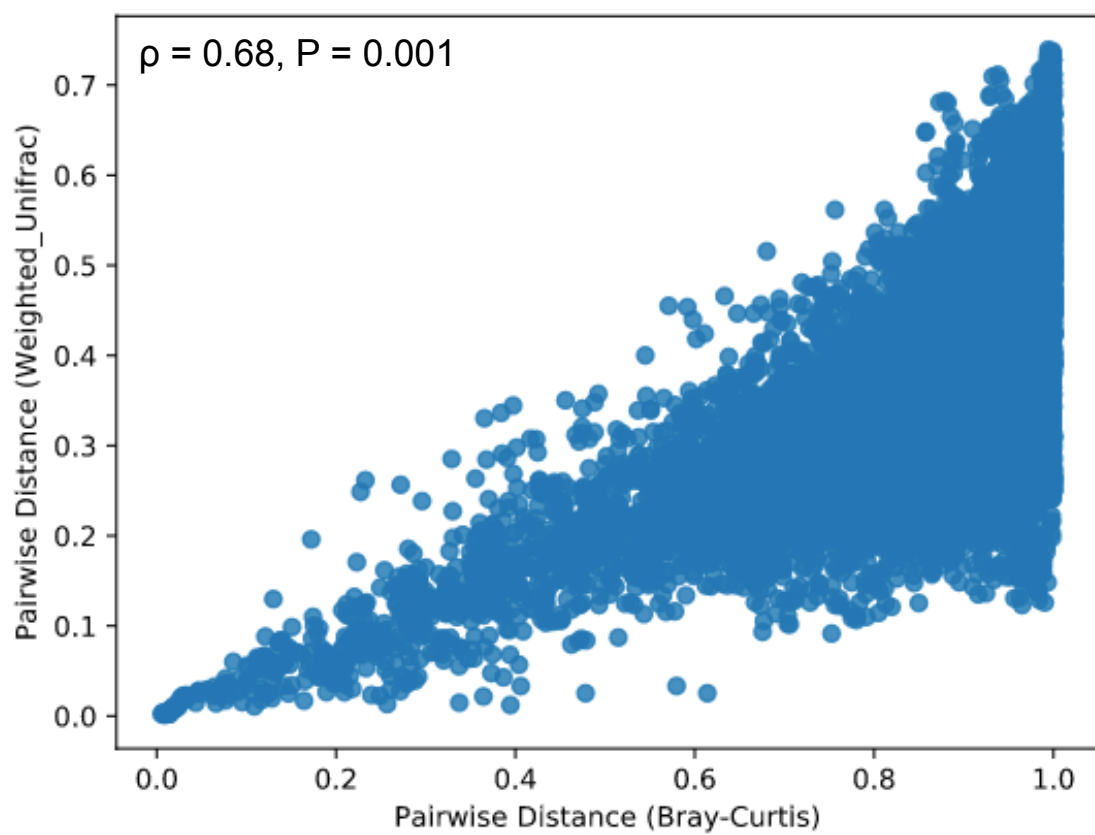

Supplement: FIG S1 [file sph003182568sf1.pdf]

Figure S2

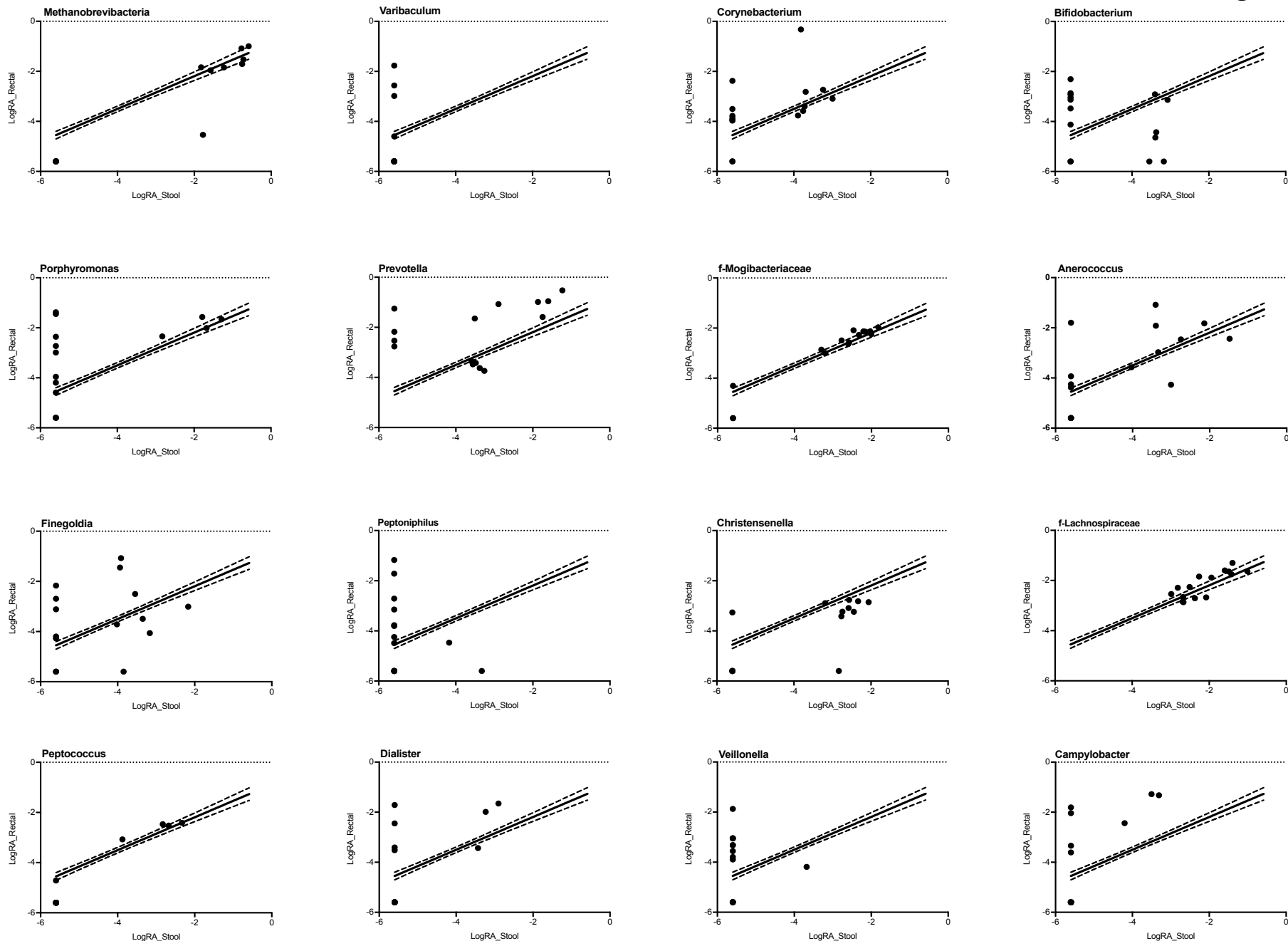

Figure S2

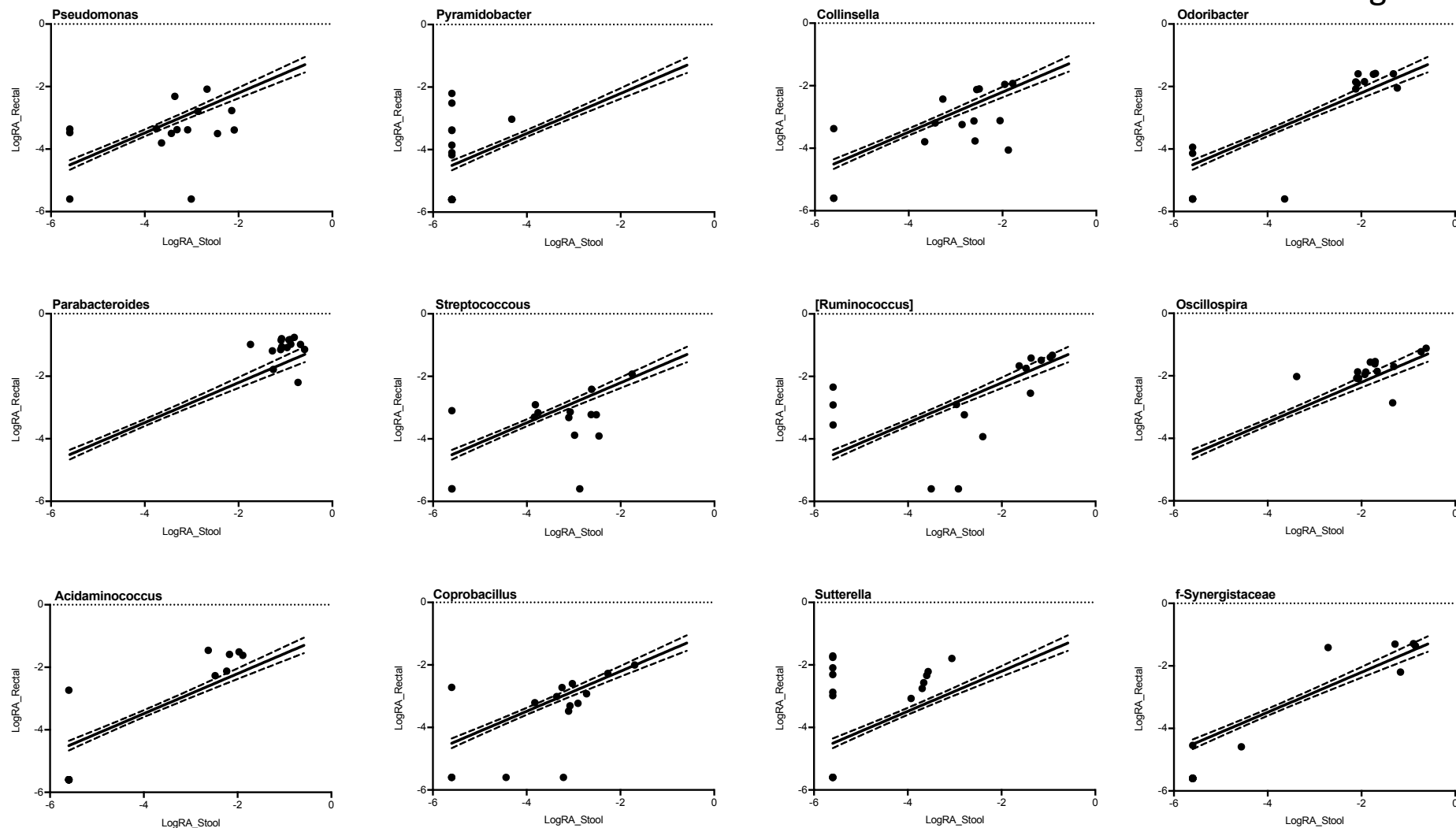

Supplement: FIG S2 [file sph003182568sf2.pdf]

Figure S3

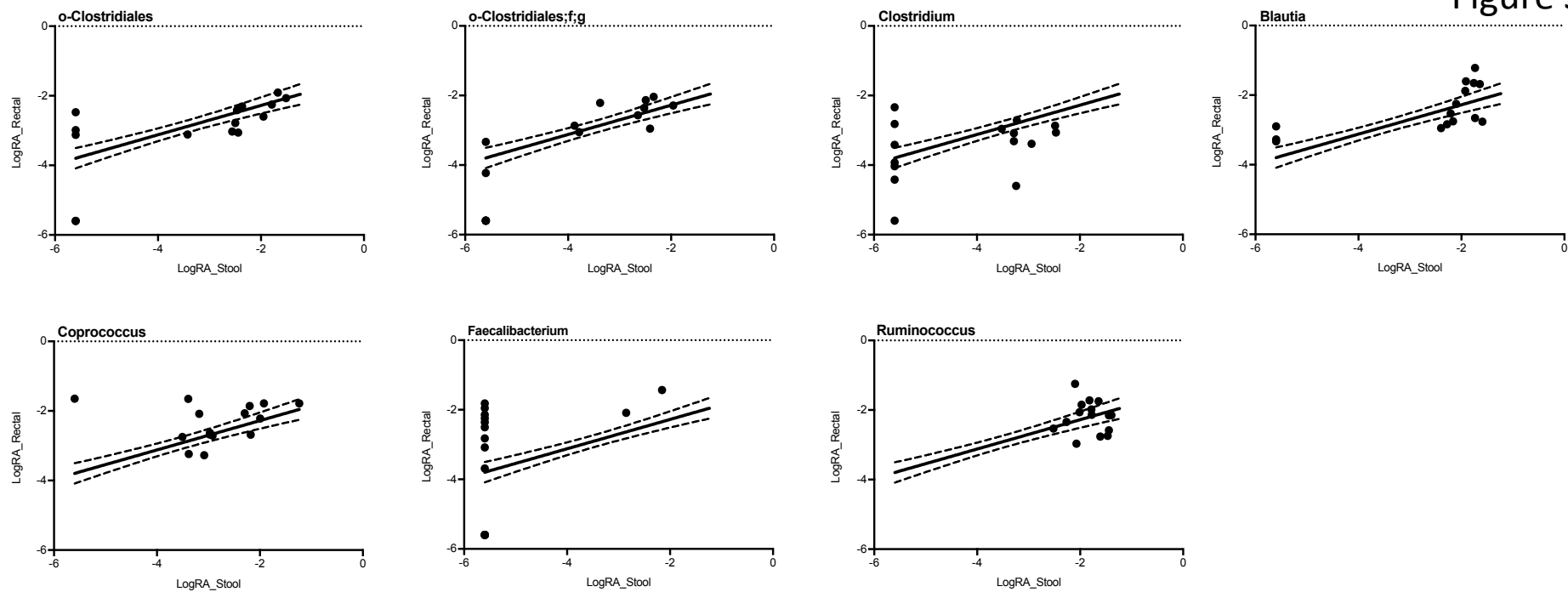

Supplement: FIG S3 [file sph003182568sf3.pdf]

Figure S4

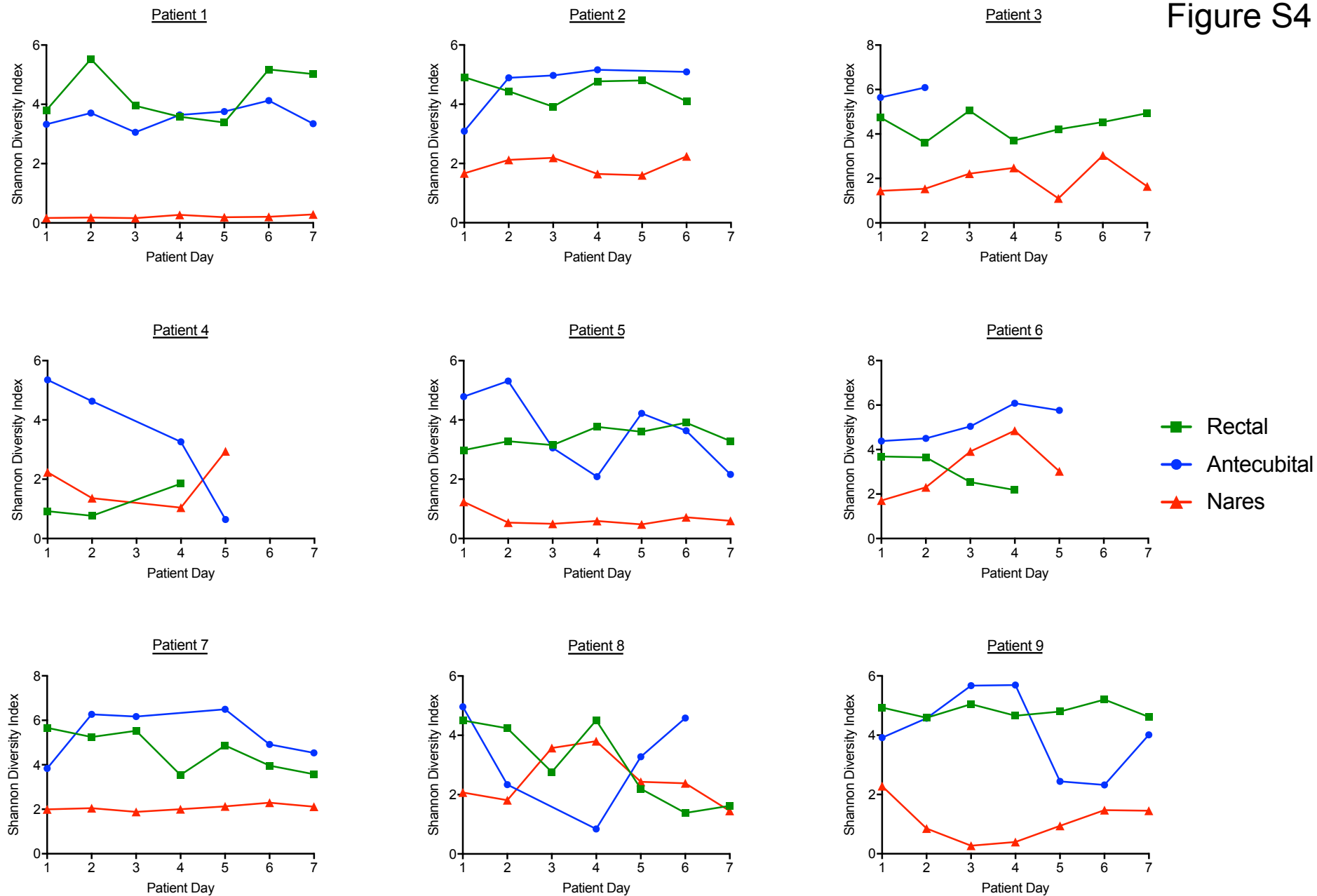

Supplement: FIG S4 [file sph003182568sf4.pdf]

Figure S5

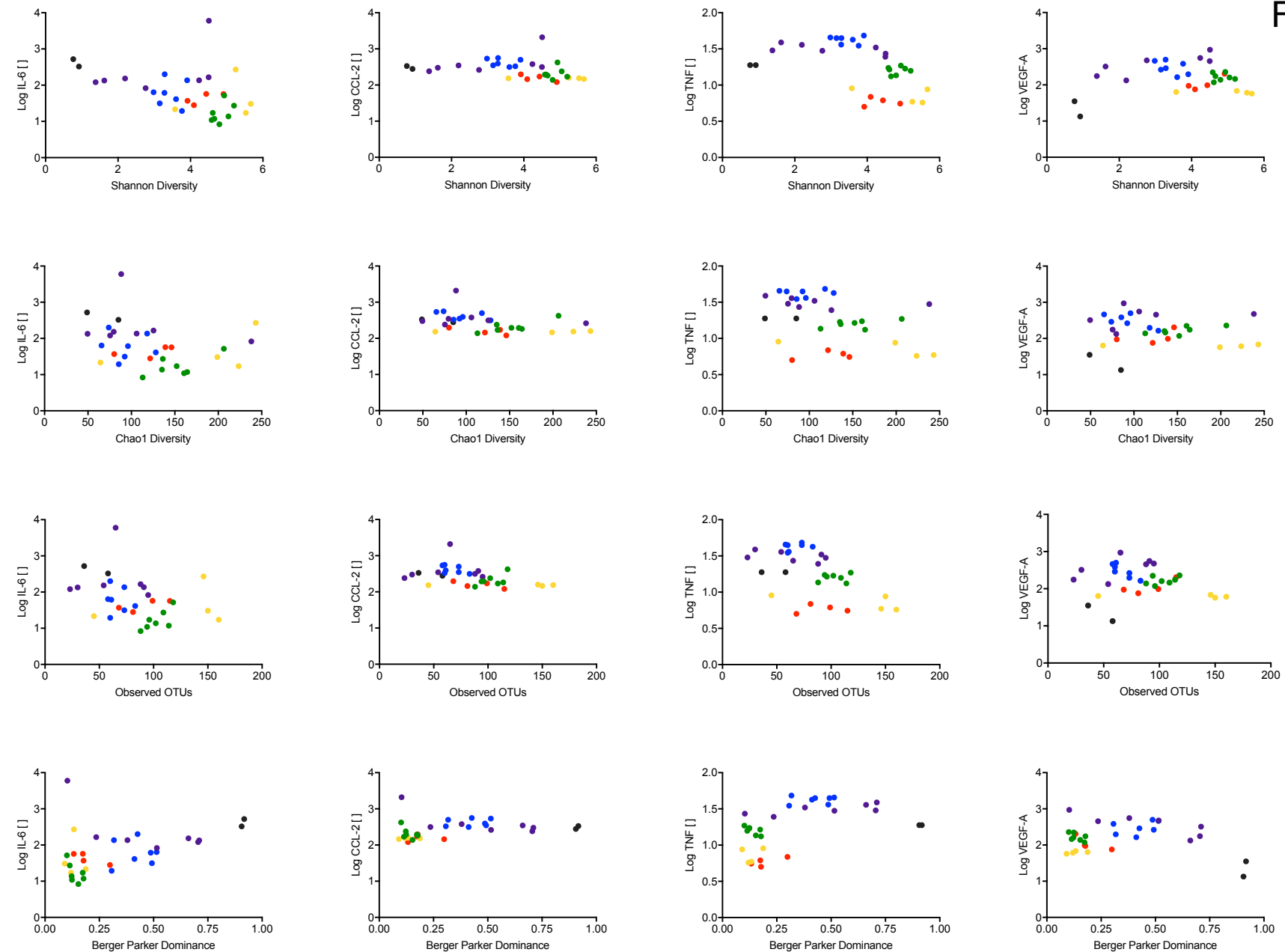

Supplement: FIG S5 [file sph003182568sf5.pdf]

Figure S6

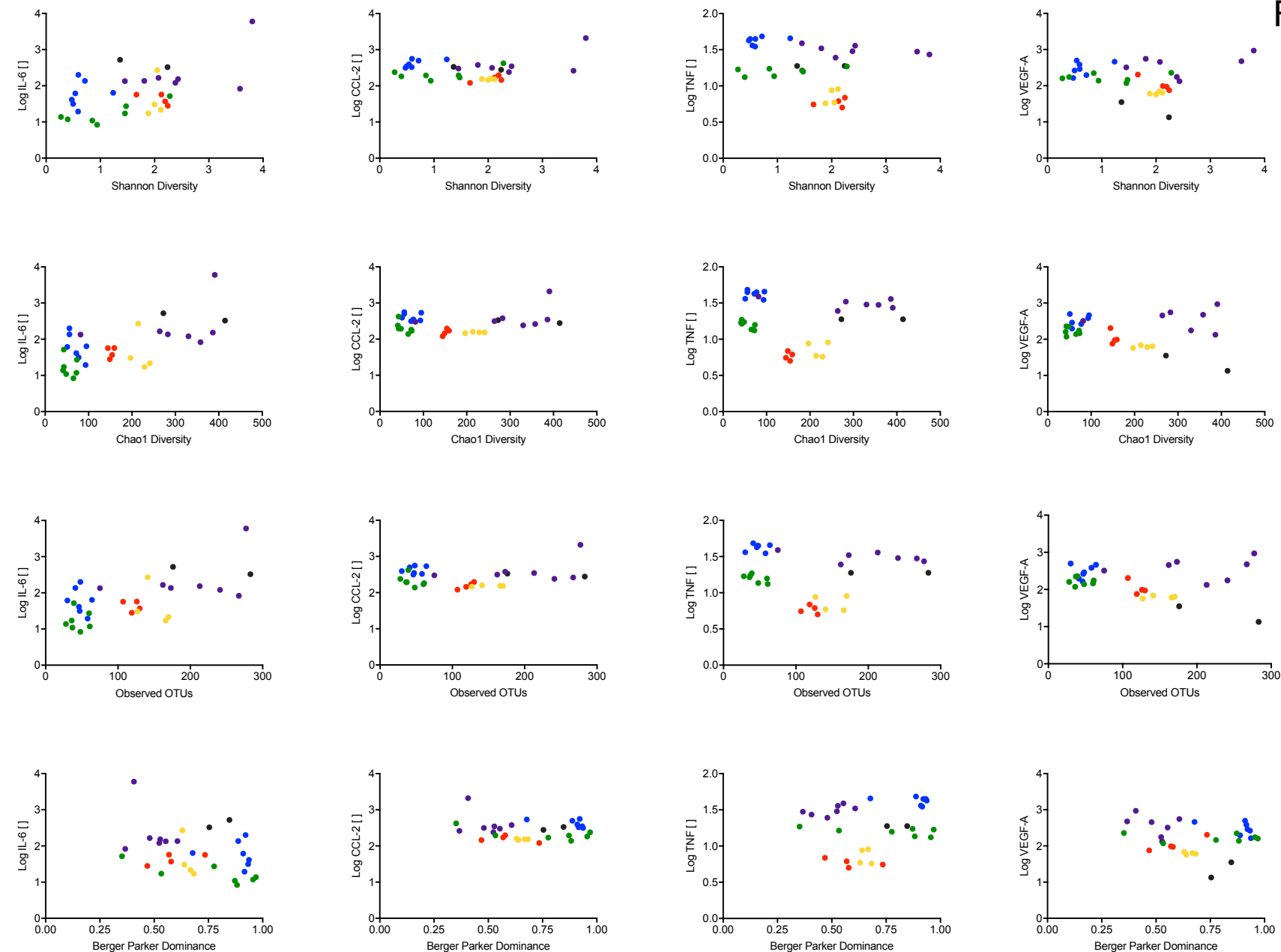

Supplement: FIG S6 [file sph003182568sf6.pdf]

**A** Gram-Staining Data

Figure S7

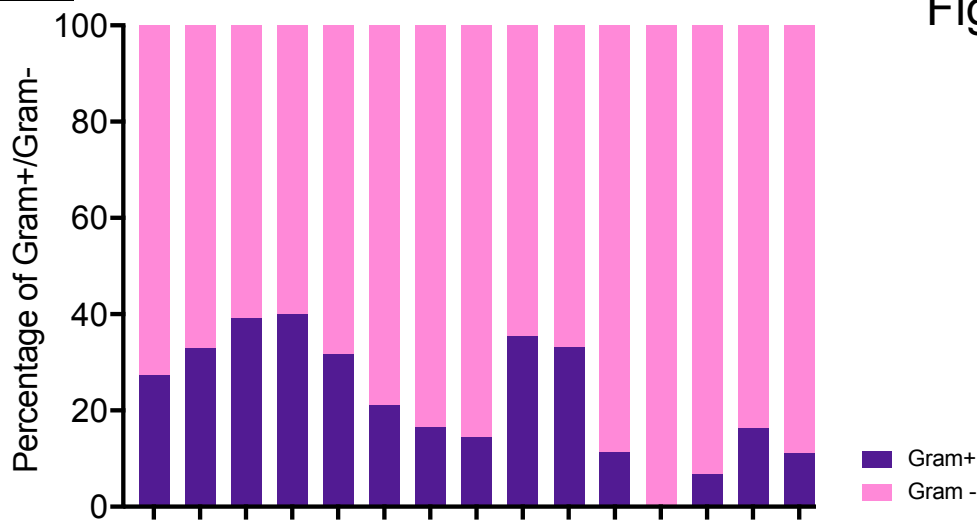

**B** 16S Gram-Assignment

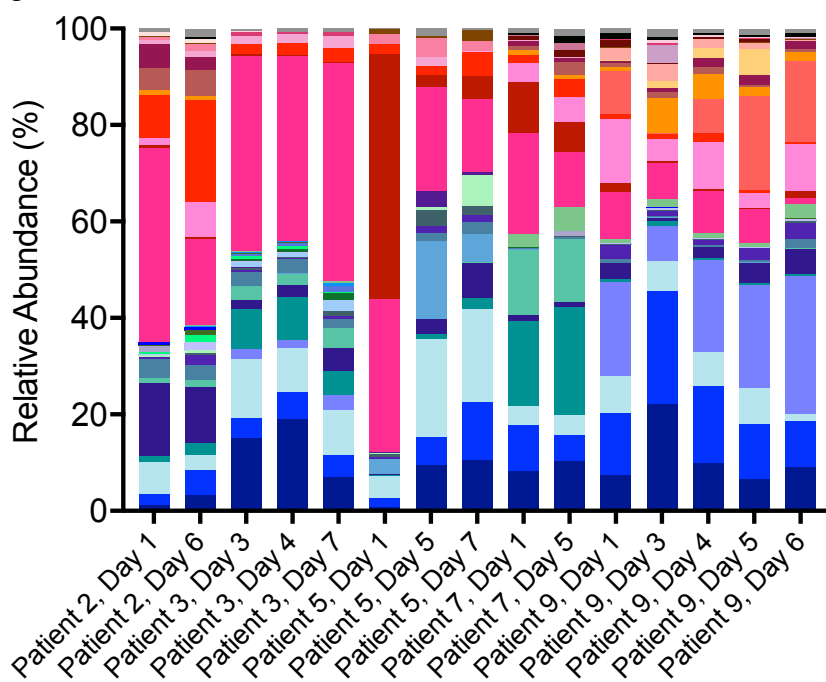

Gram-Positive

Gram-Negative

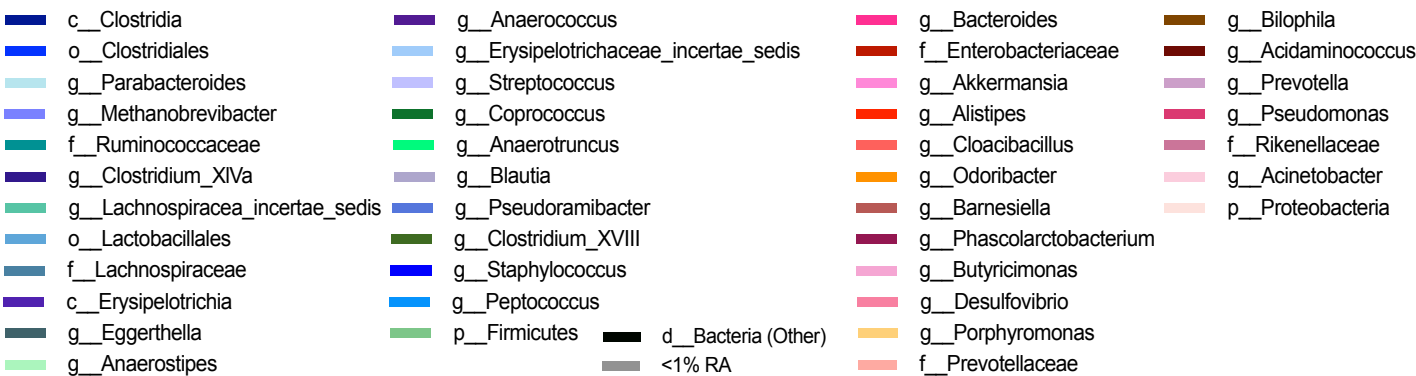

Supplement: FIG S7 [file sph003182568sf7.pdf]
