# Supplementary material for: Rectal and Naris Swabs: Practical and Informative Samples for Analyzing the Microbiota of Critically Ill Patients
Source: mSphere. 2018 Jun 13;3(3):e00219-18. doi: 10.1128/mSphere.00219-18 (PMC6001609; doi:10.1128/mSphere.00219-18)
Supplement: TABLE S1 [file sph003182568st1.pdf]

Table S1

**Bray-Curtis PERMANOVA**	Group 1	Group 2	Pseudo F value	p-value
	Antecubital	Nares	10.6	0.001
	Antecubital	Rectal	14.6	0.001
	Antecubital	Stool	12.9	0.001
	Nares	Rectal	14.5	0.001
	Nares	Stool	10.8	0.001
	Rectal	Stool	**2.8**	**0.001**
**Bray-Curtis ANOSIM**	Group 1	Group 2	R-value	p-value
	Antecubital	Nares	0.11	0.001
	Antecubital	Rectal	0.39	0.001
	Antecubital	Stool	0.82	0.001
	Nares	Rectal	0.50	0.001
	Nares	Stool	0.49	0.001
	Rectal	Stool	**-0.03**	**0.654**
**Unifrac PERMANOVA**	Group 1	Group 2	Pseudo F value	p-value
	Antecubital	Nares	15.1	0.001
	Antecubital	Rectal	31.4	0.001
	Antecubital	Stool	26.3	0.001
	Nares	Rectal	47.3	0.001
	Nares	Stool	27.8	0.001
	Rectal	Stool	**3.2**	**0.014**
**Unifrac ANOSIM**	Group 1	Group 2	R-value	p-value
	Antecubital	Nares	0.21	0.001
	Antecubital	Rectal	0.44	0.001
	Antecubital	Stool	0.67	0.001
	Nares	Rectal	0.59	0.001
	Nares	Stool	0.63	0.001
	Rectal	Stool	**-0.09**	**0.879**
